# Interactive web-based visualization and sharing of phylogenetic trees using phylogeny.IO

**DOI:** 10.1093/nar/gkz356

**Published:** 2019-05-22

**Authors:** Nikola Jovanovic, Alexander S Mikheyev

**Affiliations:** 1 Ecology and Evolution Unit, Okinawa Institute of Science and Technology Graduate University, 1919-1 Tancha, Onna-son, Okinawa, Japan; 2 Research School of Biology, Australian National University, Canberra, ACT, Australia

## Abstract

Traditional static publication formats make visualization, exploration, and sharing of massive phylogenetic trees difficult. A phylogenetic study often involves hundreds of taxa, and the resulting tree has to be split across multiple journal pages, or be shrunk onto one, which jeopardizes legibility. Furthermore, additional data layers, such as species-specific information or time calibrations are often displayed in separate figures, making the entire picture difficult for readers to grasp. Web-based technologies, such as the Data Driven Document (D3) JavaScript library, were created to overcome such challenges by allowing interactive displays of complex data sets. The new phylogeny.IO web server (https://phylogeny.io) overcomes this issue by allowing users to easily import, annotate, and share interactive phylogenetic trees. It allows a range of static (e.g. such as shapes and colors) and dynamic (e.g. pop-up text and images) annotations. Annotated trees can be saved on the server for subsequent modification or they may be shared as IFrame HTML objects, easily embeddable in any web page. The principal goal of phylogeny.IO is not to produce publication-ready figures, but rather to provide a simple and intuitive annotation interface that allows easy and rapid sharing of figures in blogs, lecture notes, press releases, etc.

## INTRODUCTION

Traditional static publishing formats struggle to display complex data sets, including large phylogenetic trees. Typically, such trees become split across multiple figures, sometimes spanning several pages, which makes them difficult to navigate, or else they are reduced in size, which makes them difficult to read. Even more problematic is that each phylogeny often contains multiple layers of information, including error bars for node date estimates, taxon images, and various other types of annotation that cannot be displayed simultaneously without crowding the figure. Consequently, the same tree may appear in multiple figures, each one displaying different aspects of the data. Spreading layers of information across multiple figures renders it difficult to see how they interrelate, and can increase publication costs, which are often based on the number of color figures.

In response to these limitations, a wide range of electronic solutions have been developed to help visualize trees, including EvolView, iTol, Icy Tree, and PhyloWidget (1–5) (See also (7) for a general review of visualization software). In addition, the phylotree.js library enables interactive trees to be included in web applications (7). These applications have rich feature sets, and produce beautifully rendered, sometimes publication-ready figures. Yet they tend to focus on visualization of the tree within the application itself, either installed locally or via a web server interface.

The phylogeny.IO web server also displays data-rich phylogenetic trees, with the goal of rendering interactive web-based visualizations. It has two modes: a fully featured annotation mode and a simplified display mode that can be embedded in most web pages.

## FEATURES

**Figure 1.**
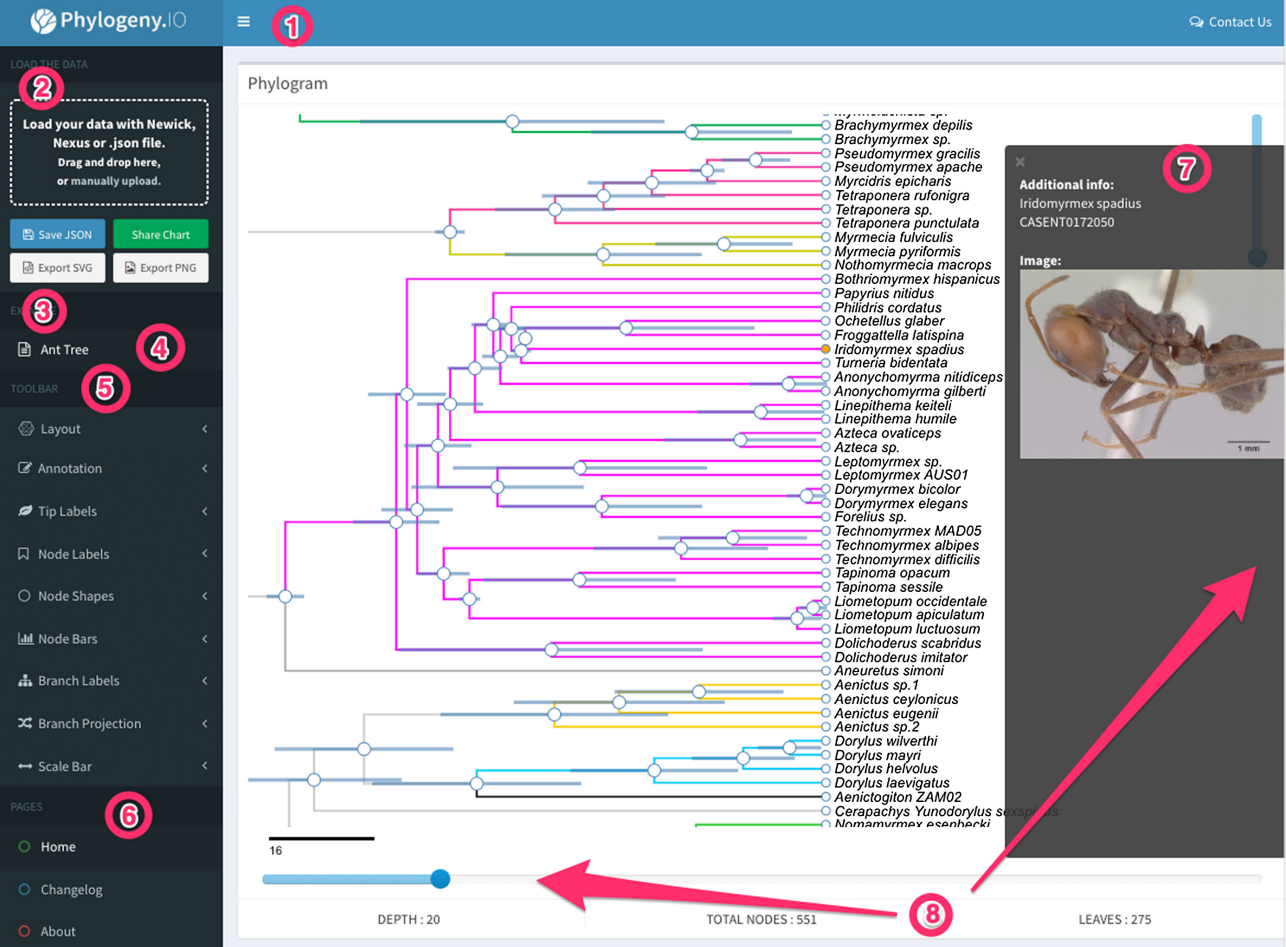
The interface of phylogeny.IO. The header bar (1) provides a point of contact for the user and displays messages about whether the tree was loaded successfully using the file import window (2). (3) There are several ways to share trees, either as graphics, or as IFRAME objects that can be embedded in HTML text. The latter feature is accessed via the ‘Share Chart’ button and allows the user to share the tree exactly as it is displayed. (4) An illustrative example quickly allows the user to become familiar with features of the site. (5) The toolbar, which has most of the options hidden by default, enables the user to annotate the tree. (6) Miscellaneous information and help pages. (7) Clicking on individual taxa pulls up additional information, including links hosted elsewhere on the internet. (8) Sliders control vertical and horizontal geometries of the tree. Image of the ant is from www.antweb.org

What sets phylogeny.IO apart from existing software is the ease with which trees can be annotated and shared. No programming experience, website maintenance, or account registration is necessary. The interface format and annotation capabilities are inspired by the popular FigTree software package (8), and incorporate most of its features (Figure 1). Resulting lightweight phylogenetic visualizations can easily be embedded into other HTML documents using IFRAME elements. As a result, interactive trees visualized in phylogeny.IO can readily be included in online course notes, blogs, press releases or even as supplements in publications.

Our goal was to develop a web server using general purpose JavaScript libraries, specifically D3.js, for phylogenetic tree display. As a result, the application works with modern browsers, including mobile devices, and allows interactive phylogenetic trees to be easily embedded in static web pages for sharing. The use of JavaScript for phylogenetic tree visualization has several advantages. First, visualization is performed client-side by the web browser, decreasing the computational load on the server hosting the trees. This enhances deployment scalability, allowing the server to handle many requests. Second, all data used to render and annotate trees are therefore accessible to the user, making the process reproducible and transparent. The web server has been available as a preprint during development and has received over ten thousand visits during this time from users around the world (9).

## EXAMPLE

**Figure 2.**
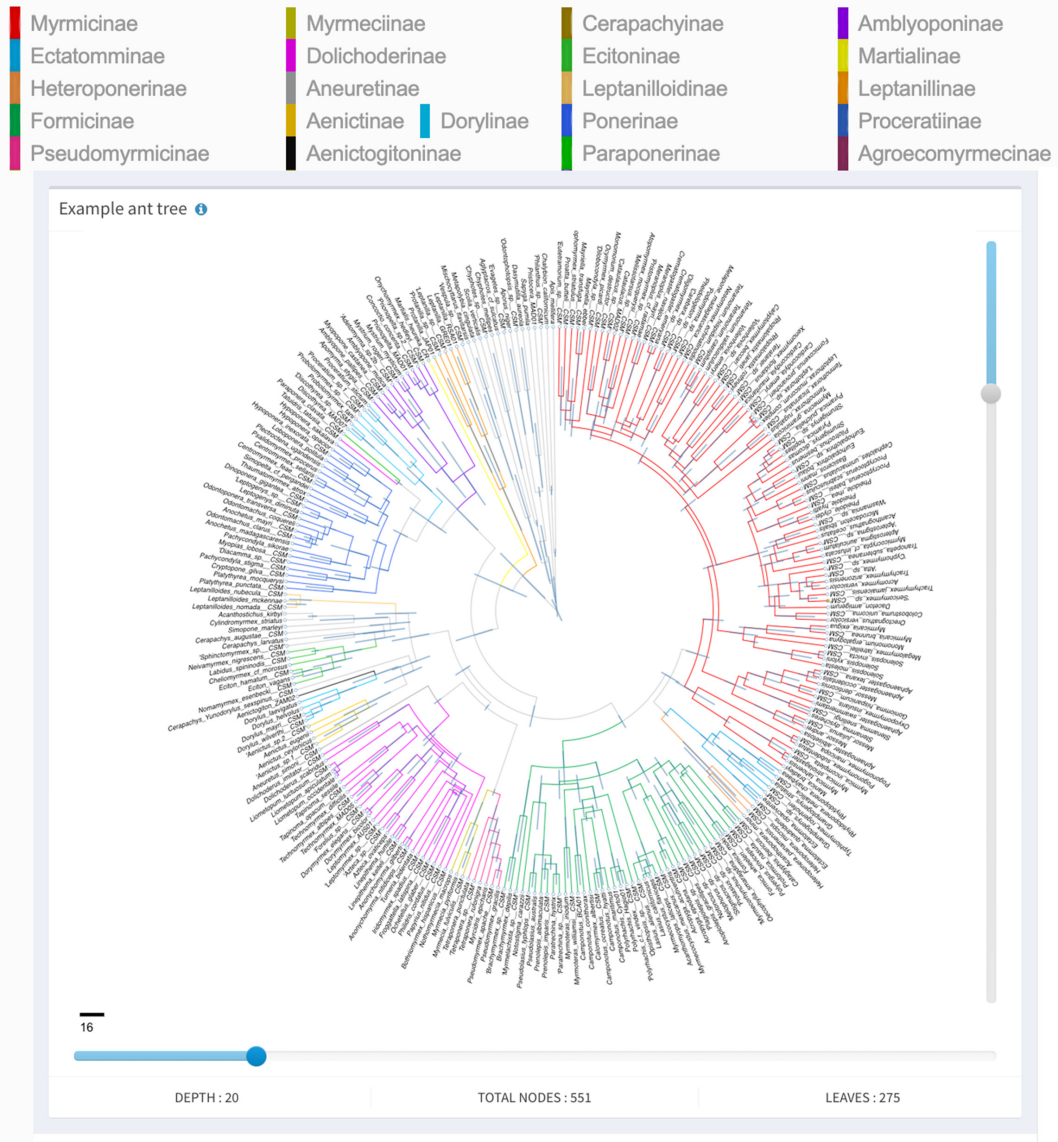
A shared tree embedded in an HTML document. The tree visualized by the web server is surrounded by a gray barrier. Everything else is the HTML web page embedding the tree. A tree description can be obtained by clicking on the letter ‘i’ of the tree. The tree is fully interactive, as it would be on the web server page, but does not have distracting toolbars and other features necessary for editing it. Trees can thus be rendered in any HTML document and even displayed on mobile devices.

We illustrate the advantages of this approach using a sizeable tree from a large study of ant phylogenetics by Moreau and Bell (10), which produced a fossil-calibrated phylogeny of 311 taxa. In the paper, the phylogeny occupies nearly three manuscript pages, together with separate figures depicting a dated chronogram with leaf labels in 1.5-point font. The annotated phylogram, combining elements of Figures 1 and 2 in the original data set can be seen as an example on the phylogeny.IO website (http://about.phylogeny.io/). In addition, where available, named species have images hyperlinked from AntWeb.

## AN INTUITIVE GRAPHICAL USER INTERFACE

For phylogeny manipulation and annotation, we borrowed heavily from the user interface of FigTree, a popular tree viewer associated with the BEAST software package (8,11) (Figure 1). Trees can be uploaded and annotated using the interface hosted at http://phylogeny.io. It currently supports Newick and NEXUS files produced by BEAST (12), PhyloXML, and a custom JSON format that is also used for exporting annotated trees. Note that non-tree blocks, such as the DATA block in NEXUS should not be included with the tree file. Once a tree has been annotated, it can be shared as an HTML iframe document and embedded into any static web page.

## AUTOMATED ANNOTATION OF FEATURES USING ANNOTATIONS WITHIN THE TREE

The current parser is mostly compatible with the NEXUS format, as implemented in BEAST, and it allows automated annotation of extended features. This includes adding confidence intervals to nodes, as well as changing the size, color and appearance of various features using extended data in the tree file. For a more complete description of these features, please see the screencast (http://about.phylogeny.io/#Screencast).

## TREE SHARING

The most important feature of phylogeny.IO is its ability to share trees that can be embedded in standard HTML documents. This is illustrated in the online documentation, which embeds the example tree (http://about.phylogeny.io/#Share) (Figure 2). In this case, the surrounding HTML provides information such as the color-coded legend, while the tree itself is retrieved from the web server.

## COMPUTATIONAL LIMITATIONS

One disadvantage of JavaScript for visualizing large data sets is that images have to be rendered in web browsers locally. This limits the size of the tree that can be visualized without slowing down the browser. As a result, the visualization is currently limited to 4000 leaf nodes total, though only 1000 leaves can be displayed simultaneously. Consequently, when larger trees are loaded, some branches are automatically collapsed to speed up rendering. In practice, we do not imagine that many users will share such large trees embedded in HTML documents, since explaining them in the actual document would be impossibly complicated, and the capacity of the user to interact with thousands of taxa would be limited.

## CONCLUSION

Phylogeny.IO allows users to easily implement a wide range of phylogenetic visualizations, and to share them with readers in any HTML document. The web server will be housed using state-of-the-art infrastructure at the Okinawa Institute of Science and Technology Graduate University, which will allow the site to scale with any increases in computational demand and popularity. Documentation for using the site is available on http://about.phylogeny.io/, including a screencast providing an overview of the project's goals and basic functionality, to provide an easy way to annotate trees and embed them in other web-based documents.

## FUTURE DIRECTIONS

We intend to keep phylogeny.io up-to-date and to incorporate suggestions from users, who can submit suggestions and bug reports via the ‘Contact Us’ link in the upper right corner of the site (Figure 1). Furthermore, errors are automatically logged and inspected by us in order to improve site functionality. Future development will focus on improving NEXUS format compatibility and addition of more standardized formats, such as a more complete implementation of NHX and being able to import PhyloXML (13). While most of the code base, excluding the server backend, exists as open source (https://github.com/oist/phylogeny-io), we would ultimately like to convert future development of the phylogeny.io web server into a community project. The back end can easily be installed on a PHP server, and allows users to modify the code and to add their own functionality, submitting pull requests for inclusion on the main server.
